# Cement-Based Wave-Absorbing Materials: A Bibliometric-Informed Critical Review

**DOI:** 10.3390/ma19214542

**Published:** 2026-10-28

**Authors:** Yue Tian, Yonggang Deng

**Affiliations:** 1 School of Material Science and Engineering, Shenyang Ligong University, Shenyang 110159, China; 2405620563@stu.sylu.edu.cn; 2 School of Intelligent Construction and Environmental Engineering, Shenyang Urban Construction University, Shenyang 110167, China

**Keywords:** cement-based materials, microwave-absorbing concrete, electromagnetic interference shielding, reflection loss, knowledge mapping, CiteSpace

## Abstract

Cement-based wave-absorbing materials have attracted rapidly growing attention because they integrate electromagnetic (EM) protection with load-bearing capacity, durability, and fire resistance. However, existing bibliometric studies remain largely descriptive and rarely link publication patterns to mechanistic advances, design strategies, or engineering bottlenecks. This study aims to map the knowledge landscape of cement-based wave-absorbing materials and identify research gaps. It further links bibliometric patterns to engineering bottlenecks. A total of 292 Web of Science Core Collection records were analyzed. Publication output increased exponentially (R^2^ = 0.92), with three stages: embryonic exploration (2015–2018), steady growth (2019–2022), and rapid expansion (2023–31 July2025). China led with 175 papers (60.0%), but international collaboration remained fragmented (network density = 0.0521). Keyword co-occurrence and burst analyses revealed a transition from admixture and matrix modification to carbon/magnetic filler engineering, porous and multilayer architectures, 3D printing, hydration products, and microstructure regulation. Drawing on a critical synthesis of representative studies, this review proposes three knowledge gaps: (i) weak coupling between EM-loss mechanisms and advanced forming processes; (ii) insufficient attention to low-frequency performance and long-term durability; and (iii) limited international standardization. Representative performance data show that porous foams can achieve an effective absorption bandwidth (EAB) up to 14.8 GHz but often with compressive strength <10 MPa, whereas pyramidal architectures can maintain >40 MPa with broadband absorption. Future research should prioritize AI-guided inverse design, cement-specific additive manufacturing, durability-oriented standardization, and sustainable material cycles. This bibliometric-informed critical review provides a data-driven roadmap for translating laboratory absorbers into engineering applications.

## 1. Introduction

The rapid advancement of information technology and the widespread deployment of electronic devices have profoundly reshaped human activities, while simultaneously raising increasingly serious concerns over electromagnetic (EM) pollution [1,2]. Excessive EM radiation has been associated with potential health risks to the public [3], and it also poses a serious threat to the operational reliability of military communication and electronic systems [4]. As building envelopes serve as primary interfaces with the external EM environment, cement-based materials are widely used in civil and defense construction, owing to their superior mechanical properties, durability, fire resistance, low cost, and widespread availability [5]. However, plain cement-based materials inherently exhibit poor EM impedance matching and negligible wave attenuation, rendering them inadequate for meeting modern EM protection requirements [6]. Therefore, enhancing wave-absorbing performance through composition regulation and structural design—and thereby achieving the integration of structural and EM shielding functions [7,8]—has become a key research priority, offering both theoretical value and practical engineering significance. From an engineering perspective, the significance of this topic lies in the growing demand for electromagnetic protection in building envelopes, defense infrastructure, and dense urban environments. In these applications, cement-based materials are used at large scale. Systematic industrial production statistics for wave-absorbing cementitious products are not available in the bibliometric literature. However, sustained growth in publication output, an increasing number of engineering-oriented studies, and the emergence of 3D-printed and prefabricated demonstrators indicate that this is a technologically significant issue rather than a purely academic one.

Current efforts to enhance the wave-absorbing performance of cement-based materials primarily focus on two technical pathways. The first is multi-component composite modification, which involves incorporating various absorbers into the cement matrix to optimize mixture proportions [9] and synergistically tailor both dielectric and magnetic loss properties. Representative modifier systems include carbon-based resistive loss materials (e.g., graphene and carbon nanotubes) [10], dielectric loss materials, such as carbon-black-modified foam cementitious systems [11], and magneto-dielectric materials (e.g., ferrites and metal oxides) [12,13]. The development of hybrid absorbers, such as ferrite-carbon fiber composites, has emerged as a key strategy for achieving synergistic magnetic-dielectric loss. The second pathway is structural design optimization, which improves wave-absorbing performance by tailoring the material’s architecture through approaches such as porous structures, multi-layer configurations, or metamaterial designs [14,15,16]. Therefore, systematically mapping the research landscape and identifying current research hotspots and technological bottlenecks can not only improve our understanding of development trends but also provide theoretical support and practical guidance for promoting the large-scale application of these materials in EM protection engineering.

Given the vast volume of published literature, it is challenging for researchers to discern the global research landscape or accurately identify frontier topics using traditional qualitative review approaches, which have inherent limitations. A preliminary search was performed in the Web of Science Core Collection on 31 July 2025, combining the topic terms with “bibliometric” OR “CiteSpace” OR “knowledge mapping.” The search returned no bibliometric review focused specifically on cement-based wave-absorbing materials. Existing reviews in this field remain predominantly qualitative and focus on specific material systems or preparation techniques. They lack a data-driven mapping of publication patterns, collaboration networks, and thematic evolution. Bibliometric analysis has become increasingly common across diverse research fields, including ceramics [17], methodological guidance [18], tourism [19], hospitality [20], green roofs [21], and green buildings [22]. However, no bibliometric review has systematically examined cement-based wave-absorbing materials. By contrast, the present study focuses specifically on cement-based wave-absorbing materials and seeks to reveal research trends related to composition modification, structural design, and EM wave absorption mechanisms. This complementary perspective helps to clarify the development trajectory of cement-based functional materials from a structure–function integration standpoint.

This study therefore moves beyond descriptive bibliometrics and adopts a bibliometric-informed critical review approach. In this review, bibliometric analysis serves as a mapping tool to identify research clusters and gaps. The critical synthesis of representative materials-science studies provides the main technical conclusions. The emphasis is therefore on materials strategies and engineering trade-offs, rather than on bibliometric description as such. It addresses four questions: (1) What are the publication trends, collaboration patterns, and disciplinary characteristics of this field? (2) Which research themes and hotspots have emerged, and how are they clustered? (3) How have research foci evolved over time? (4) What knowledge gaps exist among material formulation, forming processes, EM mechanisms, and long-term durability? By answering these questions, this study provides a data-driven knowledge map and a critical synthesis of mechanism–design–performance–durability linkages. A multi-scale perspective is used as a qualitative lens to bridge laboratory research and engineering application. Ref. [6] proposed a multi-scale design framework for cementitious absorbers from a materials-design perspective, focusing on mechanism-based material and structure design. The present study differs in scope and method. It is bibliometric-informed, mapping publication and collaboration patterns, identifying cluster-level knowledge fragmentation, and linking bibliometric clusters to performance trade-offs and engineering bottlenecks. Here, the multi-scale perspective serves as a qualitative lens that organizes the critical synthesis, rather than as a newly proposed design methodology.

## 2. Methods and Analytical Framework Based on CiteSpace

### 2.1. Data Source

Data were retrieved from the Web of Science Core Collection (SCI-Expanded) on 31 July 2025. The search covered the period from 2015 to 31 July 2025; therefore, 2025 is treated as a partial year, and only English-language publications were included. The search string was as follows:

TS = ((“cement-based” OR cementitious OR cement) AND (“wave-absorbing” OR “microwave absorption” OR “electromagnetic absorption” OR “electromagnetic shielding” OR “EM absorption”) AND (composite* OR modified OR filler* OR admixture*)).

The search was refined to the following Web of Science categories: Construction & Building Technology, Materials Science Multidisciplinary, Engineering Civil, and Engineering Environmental. These categories were selected because they cover the core journals that publish cement-based wave-absorbing studies. Categories such as Physics Applied and Materials Science Ceramics were excluded because they are dominated by non-cement systems. This exclusion may omit a small number of relevant records, which is acknowledged as a limitation. Document types were restricted to Article and Review Article. “Electromagnetic shielding” was included only to capture studies reporting both absorption and shielding performance. Shielding-only studies were retained only when they offered mechanistic or design insights relevant to absorption. The initial search yielded 412 records. The 47 duplicates arose mainly from overlapping online-first and final records, as well as from metadata variants.

After de-duplication, two reviewers independently screened titles and abstracts using five predefined criteria: (i) non-cement systems; (ii) no electromagnetic absorption or shielding performance; (iii) conference abstracts, editorials, or book chapters; (iv) non-English records; and (v) studies focused exclusively on shielding without absorption-related discussion. Shielding-only studies were retained only when they provided absorption-related mechanistic or design insights. Disagreements were resolved by discussion. To assess inter-rater agreement, a random sample of 30 records was independently double-screened; Cohen’s kappa was 0.78 (substantial agreement).

Although the search was restricted to English-language publications and to Article/Review Article document types, 8 non-English and 12 conference/editorial records still appeared in the export because Web of Science language and document-type tags are sometimes incomplete or inconsistently assigned. These records were removed at screening. The 47 duplicates were identified by matching titles, authors, and DOIs. They arose mainly from overlapping online-first and final records and from metadata variants. The 73 excluded records were classified by primary exclusion reason: non-cement systems (*n* = 28); no electromagnetic absorption or shielding performance (*n* = 25); conference abstracts, editorials, or book chapters (*n* = 12); and non-English records (*n* = 8). The screening process proceeded as follows: 412 records identified → 47 duplicates removed → 365 screened → 73 excluded → 292 included. The screening process is summarized in Figure S1 (simplified PRISMA 2020 flow diagram). The Web of Science Core Collection was selected because it provides consistent indexing and citation coverage for construction-materials research and has been widely used in bibliometric studies of cementitious materials. Scopus was not included, and this is acknowledged as a limitation in Section 4.2. The included records were published mainly by Elsevier, MDPI, Springer Nature, and Taylor & Francis, among others.

### 2.2. Bibliometric Measurement

Using the Web of Science retrieval results, we extracted publication years, country and institution distributions, and the author, institution, and keyword networks used in subsequent analyses. The search results were exported as “Full Record and Cited References” into CiteSpace 6.4. Visualization analysis was then performed on the authors and keywords, generating the corresponding author co-occurrence networks and keyword co-occurrence clustering maps. This workflow enabled reproducible mapping of collaboration patterns and research themes in the retrieved literature.

Analyses were performed using CiteSpace 6.4 with time slicing from 2015 to 2025 (1 year per slice). The node selection criterion was the g-index (k = 25), with LRF = 2.5, L/N = 10, LBY = 5, and e = 1.0. The pruning method was the minimum spanning tree, and counts used integer counting. For node type selection, “Keyword,” “Author,” and “Institution” were selected successively to generate keyword co-occurrence, author collaboration, and institution collaboration networks, respectively. Based on these networks, the research trajectory, hotspot themes, and frontier trends in the field of cement-based wave-absorbing materials were systematically examined across multiple dimensions, including fundamental bibliometric characteristics, core author/institution collaboration patterns, keywords co-occurrence and clustering, and burst detection. This multidimensional approach provides quantitative support for both academic research and engineering applications in this field [23]. Co-word analysis assumes that frequently co-occurring keywords reflect shared research themes. Clusters were labeled using the log-likelihood ratio (LLR) algorithm, which identifies terms that are statistically overrepresented in each cluster. Burst detection followed Kleinberg’s algorithm, which identifies terms with sharp increases in frequency over a specified period. The modularity Q quantifies the strength of community structure; Q > 0.3 indicates a significant community structure. The silhouette S measures cluster homogeneity; S > 0.7 indicates reliable clustering. All network densities reported in Section 3.2, Section 3.3 and Section 3.4 refer to the pruned networks used for visualization. Unpruned densities would be higher.

### 2.3. Electromagnetic Performance Metrics and Definitions

For a metal-backed single-layer absorber, the input impedance is $Z_{in}=Z_{0}\sqrt{\frac{\mu_{r}}{\varepsilon_{r}}}tanh[j(2\pi fd/c)\sqrt{\mu_{r}\varepsilon_{r}}]$, where *Z*_0_ is the free-space impedance, *μ*_*r*_ and *ε*_*r*_ are the relative complex permeability and permittivity, *f* is frequency, *d* is thickness, and *c* is the speed of light. The reflection loss is *RL* = 20*log*|(*Z_in_* − *Z*_0_)/(*Z_in_* + *Z*_0_)|. Impedance matching requires *Z_in_* ≈ *Z*_0_, whereas attenuation is governed by the attenuation constant α. The effective absorption bandwidth (EAB) is defined as the frequency range in which RL < −10 dB. Shielding effectiveness (SE) is expressed as *SE* = *SE_R_* + *SE_A_*, where *SE_R_* and *SE_A_* denote reflection and absorption losses, respectively. In absorption-dominated materials, *SE_A_* is the dominant contribution. In this review, low frequency refers to the S- and C-band range (2–8 GHz); sub-1 GHz studies are noted separately where present.

## 3. Results and Critical Analysis

### 3.1. Publication Trends and Stage-Wise Technological Evolution

**Figure 1:**
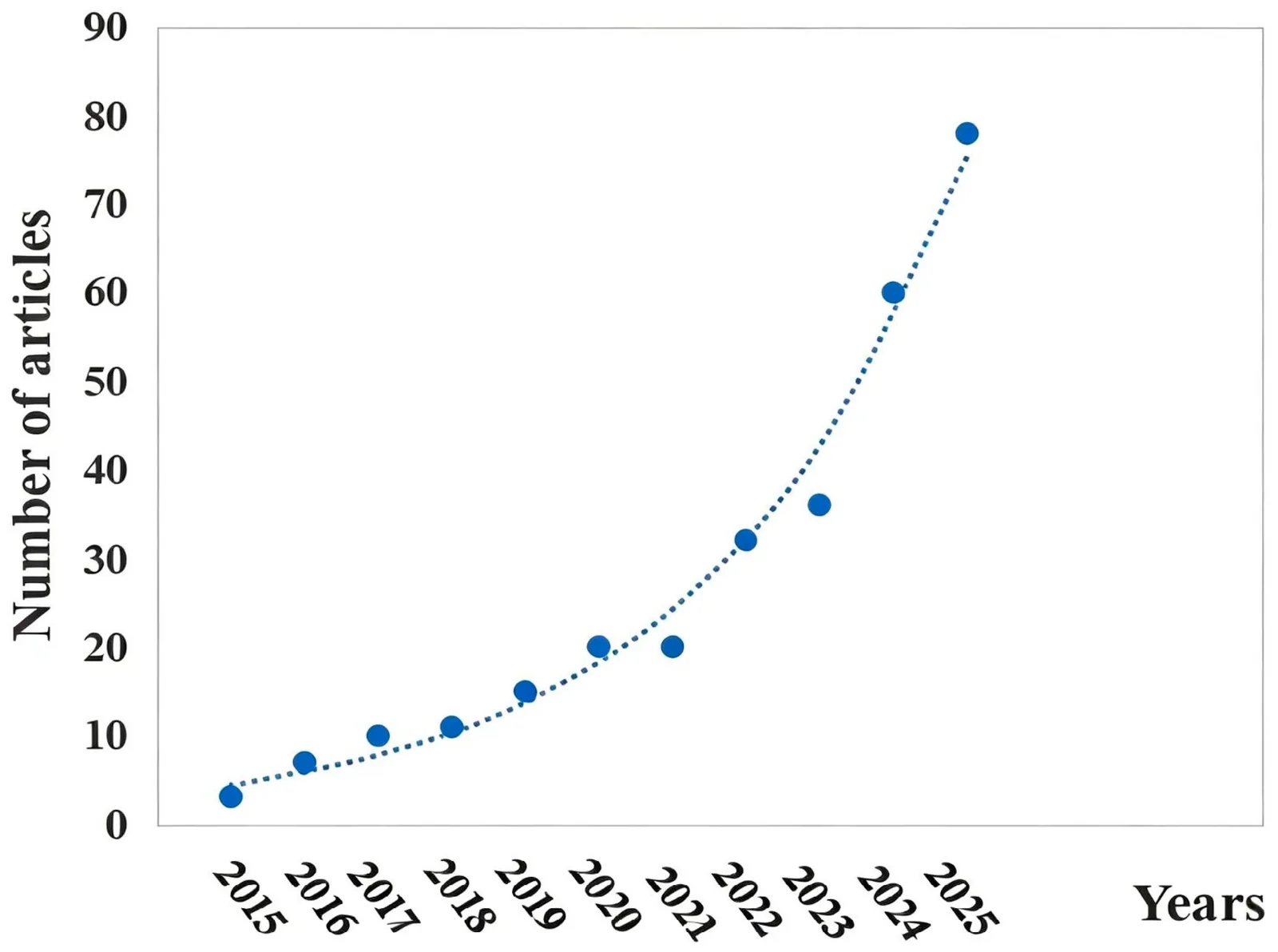
Annual distribution of publications on cement-based wave-absorbing materials (2015–31 July 2025). The bar chart shows annual publication volume; the dashed line indicates the exponential fit (R^2^ = 0.92). The three stages are embryonic exploration (2015–2018), steady growth (2019–2022), and rapid expansion (2023–).

As shown in Figure 1, the publication output from 2015 to 2025 exhibited distinct stage-wise characteristics. The three stages were defined by changes in annual publication volume, not by burst detection. Burst detection in Section 3.6 serves as a post hoc observation of keyword dynamics, rather than as validation of the stage boundaries. The first stage, embryonic exploration (2015–2018), recorded only 3–11 publications per year, indicating a period of preliminary exploration with low research attention. Research during this period primarily focused on admixture and mineral additive modification of cement matrices, as well as fundamental electromagnetic (EM) property testing [24,25,26]. A systematic research framework had not yet been established. The second stage, steady growth (2019–2022), recorded 15–32 publications per year. Driven by rising demands for EM pollution control and building shielding, research emphasis gradually shifted toward the synergistic optimization of wave-absorbing and mechanical properties. Carbon-based filler modification and solid-waste resource utilization pathways were extensively explored, while the engineering application orientation became more pronounced [27,28]. The third stage, rapid expansion (2023–31 July 2025), recorded 36–78 publications per year, with a notably steepening growth trajectory. During this stage, research expanded beyond macroscopic performance characterization to fundamental theoretical aspects, including cement hydration products, pore-structure evolution, interfacial regulation, and wave-absorption loss mechanisms [29,30,31]. The design of high-performance cement-based wave-absorbing materials featuring broadband, high-efficiency, and structure–function integration has become mainstream.

The acceleration after 2023 may be associated with several contextual factors. These include the expansion of 5G/6G communication infrastructure and increasingly complex urban electromagnetic environments; growing attention to electromagnetic protection in defense and critical infrastructure; the maturation of enabling technologies such as carbon-based and magnetic fillers and 3D printing; and increased research funding for functional construction materials. Military conflicts and urban infrastructure expansion may both contribute, but bibliometric data alone cannot disentangle their individual effects. These factors are therefore discussed as contextual interpretations rather than demonstrated drivers. Other possible influences, such as national research policies, journal special issues, and post-pandemic research recovery, cannot be excluded.

The three stages show a marked increase in publication output: from 3–11 papers per year (2015–2018) to 15–32 (2019–2022) and 36–78 (2023–31 July 2025). An exponential model fitted to the data (excluding the partial year 2025) yielded an R^2^ of 0.92. This indicates an accelerating growth trend consistent with the three-stage pattern described below. Technologically, the three publication stages corresponded to three distinct research paradigms. The embryonic stage (2015–2018) was dominated by admixture and mineral additive modification, such as fly ash and carbon black, with research focused on basic electromagnetic property testing. The main limitation was the absence of a systematic mechanism–property framework. The steady growth stage (2019–2022) shifted toward carbon-based filler modification and synergistic optimization of mechanical and wave-absorbing properties. CNTs, graphene, carbon fibers, and ferrites became representative fillers, but dispersion and impedance matching remained critical bottlenecks. The rapid expansion stage (2023–31 July 2025) moved beyond macroscopic performance characterization toward hydration products, pore-structure evolution, interfacial regulation, and multi-scale loss mechanisms. Advanced forming processes such as 3D printing and multilayer/gradient architectures also emerged as frontier topics. This stage-wise technological evolution explains why the field is now transitioning from composition-centric modification to integrated material–structure co-design. Table 1 summarizes the stage-wise technological evolution and remaining bottlenecks of this field.

### 3.2. Country/Region Distribution and Technical Specialization

From January 2015 to 31 July 2025, a total of 36 countries/regions published research on cement-based wave-absorbing materials. Among the top five countries/regions by publication volume, China ranked first with 175 papers, followed by the United States (28), India (15), South Korea (14), and Australia (13). China accounted for 60.0% of the global total. Table 2 summarizes the top five countries/regions and their main technical contributions.

**Figure 2:**
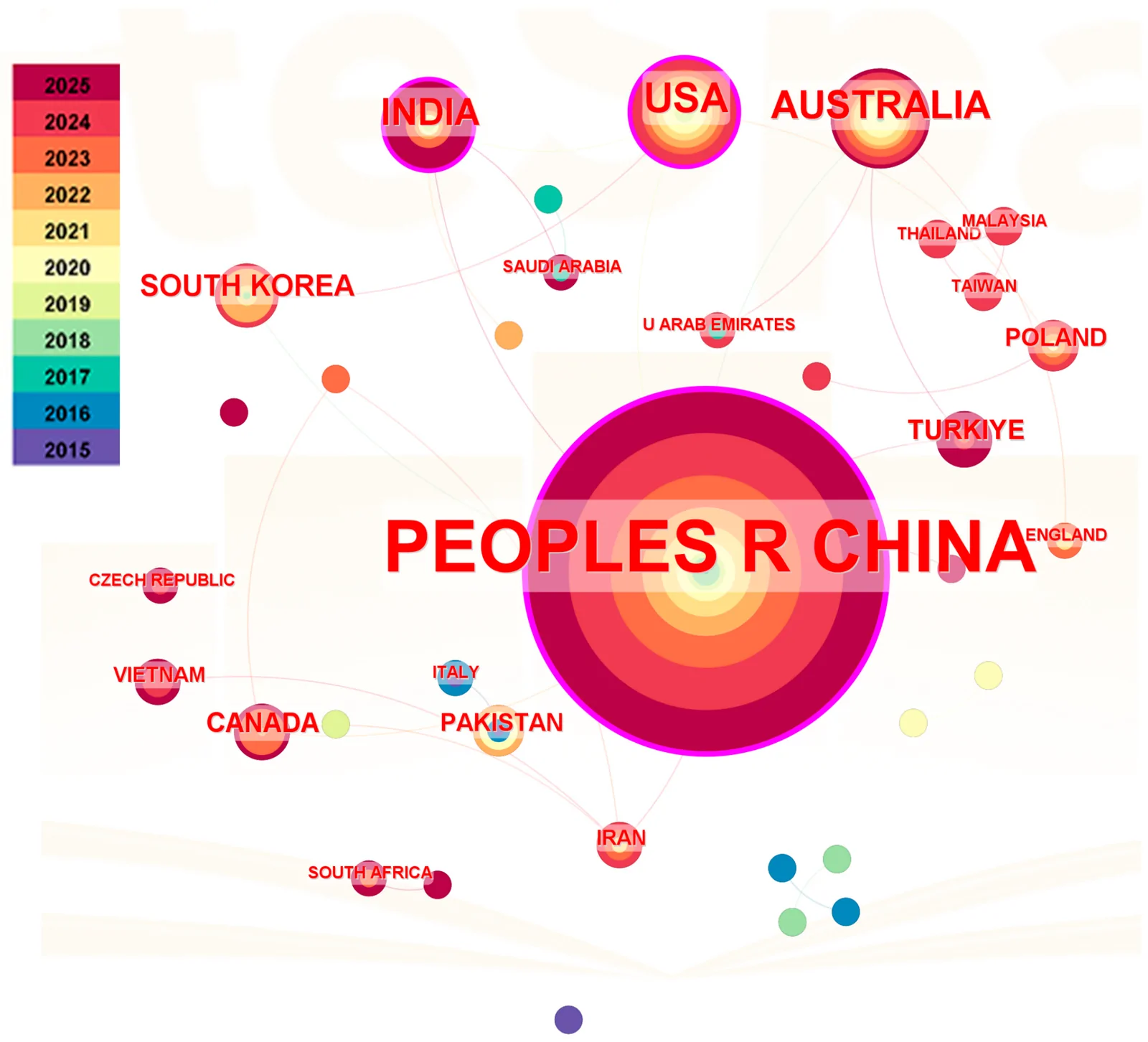
Country/region collaboration network for cement-based wave-absorbing materials research (2015–31 July 2025). Node size indicates each country’s publication share; link thickness represents international collaboration strength. The network is China-centered with dispersed multinational participation.

Keyword frequencies within each country/region group in the 292-record corpus were used to characterize representative research topics (Table 2). The country distribution also reflects different representative research topics. Chinese studies frequently address carbon-based filler modification, porous and multilayer architectures, and 3D-printed cementitious absorbers. Studies from the United States often focus on EMI shielding theory and metamaterial-inspired designs. Indian and South Korean studies frequently report ferrite-based magnetic absorbers and CNT/cement systems, respectively, while Australian studies often address 3D concrete printing and structural–functional integration. As shown in Figure 2, the country collaboration network had a density of 0.0521 for the pruned network used for visualization. Because network density depends on network size and pruning, this value is reported for descriptive purposes only and is not used as a standalone indicator of collaboration strength.

### 3.3. Institutional Distribution and Research Specialization

From 2015 to 31 July 2025, among the top 10 research institutions publishing papers on cement-based wave-absorbing materials, Hebei University of Technology ranked first with 20 publications, followed by Wuhan University of Technology (17), Dalian University of Technology (14), China Building Materials Academy (13), Shenyang Ligong University (12), University of Western Australia (11), Xi’an University of Architecture and Technology (10), Edith Cowan University (9), KAIST (8), and Southeast University (8). Table 3 summarizes the top 10 institutions and their representative research profiles.

**Figure 3:**
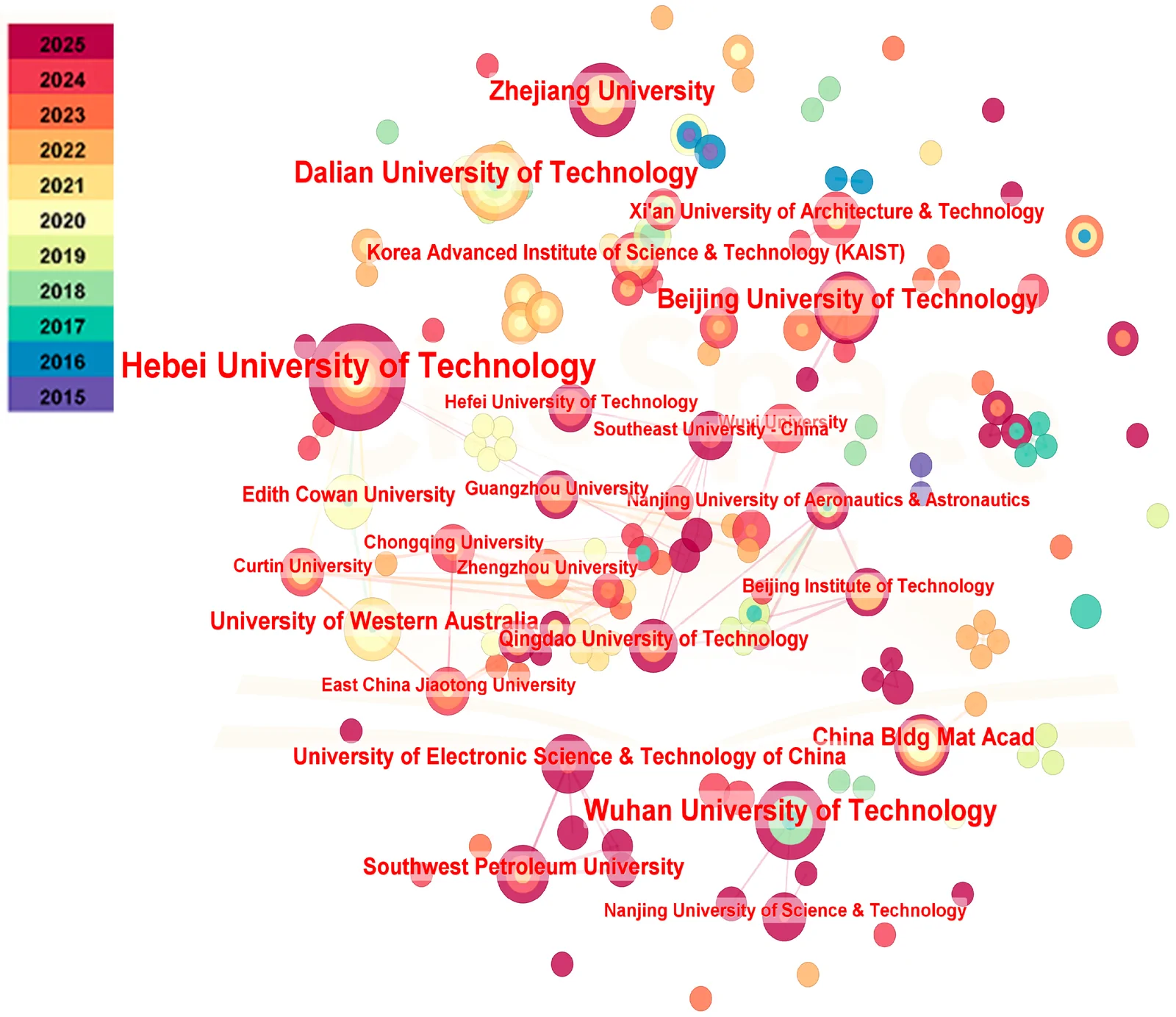
Institution collaboration network for cement-based wave-absorbing materials research (2015–31 July 2025).

Keyword frequencies within each institution group in the 292-record corpus were used to characterize the representative research foci of the top 10 institutions (Table 3). Representative work from Hebei University of Technology addresses carbon-based filler modification and electromagnetic parameter regulation [32]. Representative work from Wuhan University of Technology covers graphene-modified cement composites for electromagnetic wave absorption [33]. China Building Materials Academy and Shenyang Ligong University have published representative work on carbon black/cement systems and ferrite/graphite hybrid absorbers, respectively [34,35]. The University of Western Australia and Edith Cowan University have collaborated with Chinese institutions on 3D concrete printing for EMW-absorbing cementitious materials [36]. The ranking should therefore be interpreted as an indicator of publication volume rather than of research quality or impact. The representative keywords indicate that the top institutions are not evenly distributed across technical directions: some focus on carbon-based fillers, while others focus on magnetic fillers, 3D printing, or multilayer architectures. As shown in Figure 3, this value is reported for descriptive purposes only and is not used as a standalone indicator of collaboration strength. This distribution of representative research foci helps explain why the keyword clusters are locally dense but globally fragmented.

### 3.4. Author Collaboration and Team-Level Technological Routes

Author names were disambiguated using affiliation, co-author lists, and email domains. Common names such as “Wang, Jing” were manually checked against affiliation records. Records that clearly belonged to different research groups were separated. Residual uncertainty remains for authors with identical names and similar affiliations.

**Figure 4:**
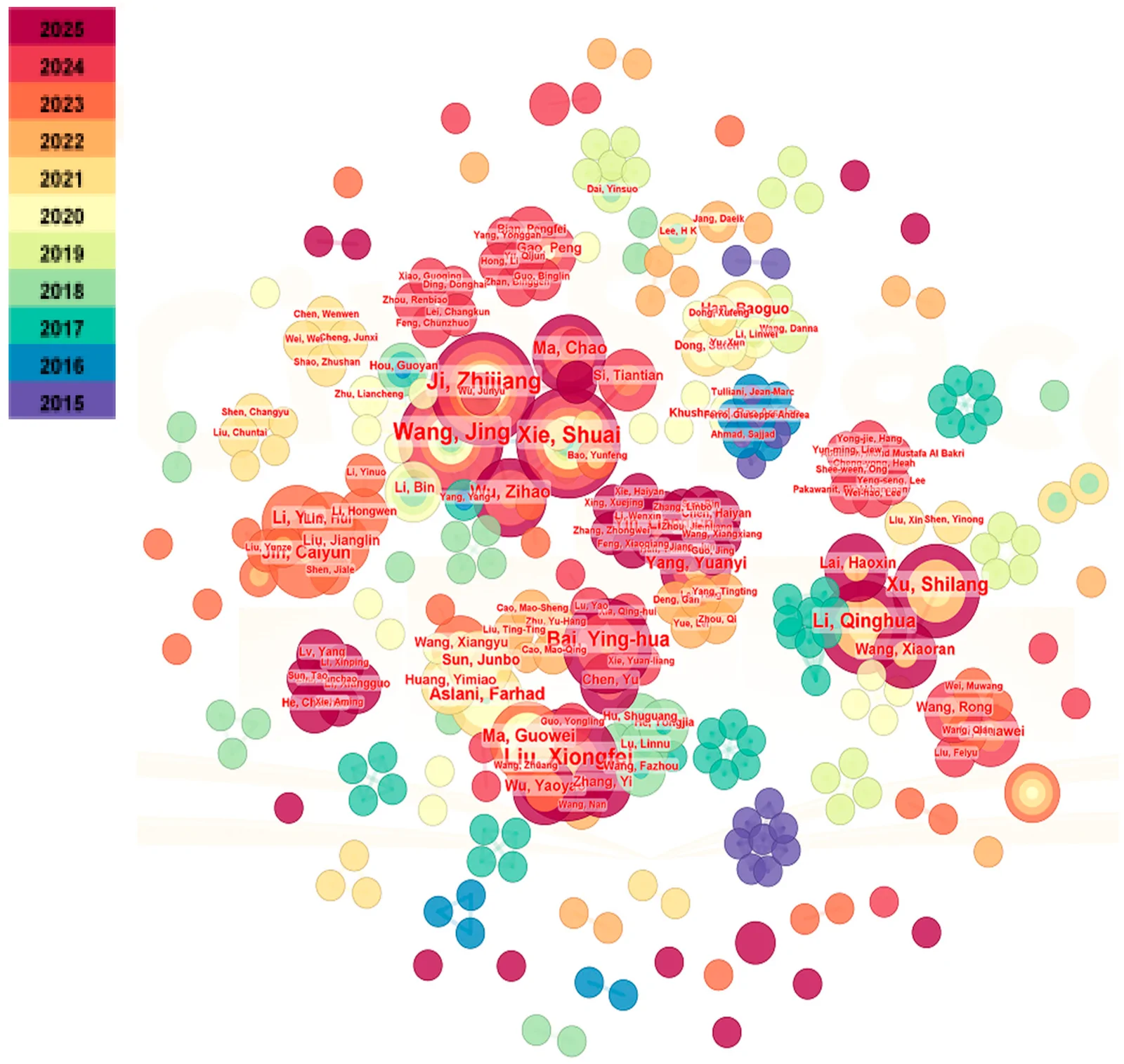
Author collaboration network. Node size indicates publication volume; link thickness indicates collaboration strength. The network density is 0.0131, indicating “global dispersion with local clustering”.

Based on the author co-occurrence network, the team led by Wang Jing and Ji Zhijiang has the highest publication output in this field, with 15 articles each. Their research focuses on carbon-based filler modification and the electromagnetic wave absorption properties of cement-based composites, including carbon black, CNTs, and graphene oxide [27,37]. The team led by Xu Shilang and Li Qinghua ranks second in publication volume, with research focusing on fiber hybridization, fly ash incorporation, carbonyl iron powder, and CNT-reinforced systems for ultra-wideband absorption [38,39,40,41]. An overseas research subgroup led by Aslani Farhad (University of Western Australia) has contributed to research on 3D concrete printing of EMW-absorbing cementitious materials [28]. Figure 4 shows the author collaboration network.

The author collaboration network comprises 296 nodes and 574 links, with a density of 0.0131 for the pruned network. Despite evident local clustering, the low density is consistent with weak large-scale cross-team collaboration. However, because density depends on network size and pruning, this interpretation is descriptive only and should not be treated as a standalone metric. The large node count indicates that this research field has attracted a substantial number of researchers, constituting a strong talent pool. This pattern can be described as “global dispersion with local clustering.” This indicates that knowledge exchange is active within established teams but limited across teams and countries.

### 3.5. Keyword Co-Occurrence and Cluster-Based Technical Mapping

**Figure 5:**
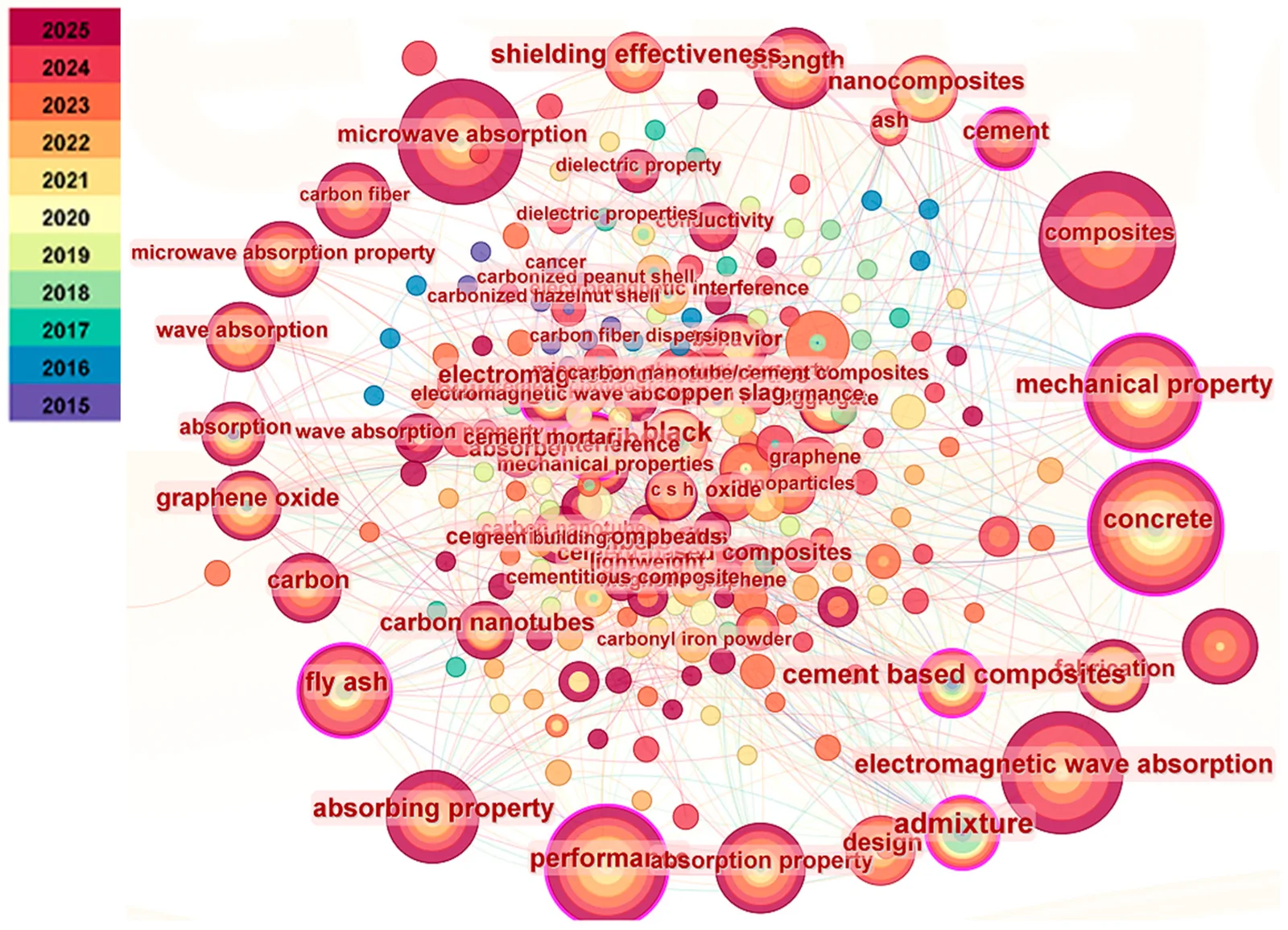
Keyword co-occurrence network of cement-based wave-absorbing materials (2015–31 July 2025). Node size reflects keyword frequency; link thickness represents co-occurrence strength. Synonymous keyword variants (e.g., microwave absorption, microwave absorption property, wave absorption, absorption, absorbing property, and absorption property; mechanical property and mechanical properties) were not fully merged in the visualization; they were treated as equivalent terms in the interpretation.

**Figure 6:**
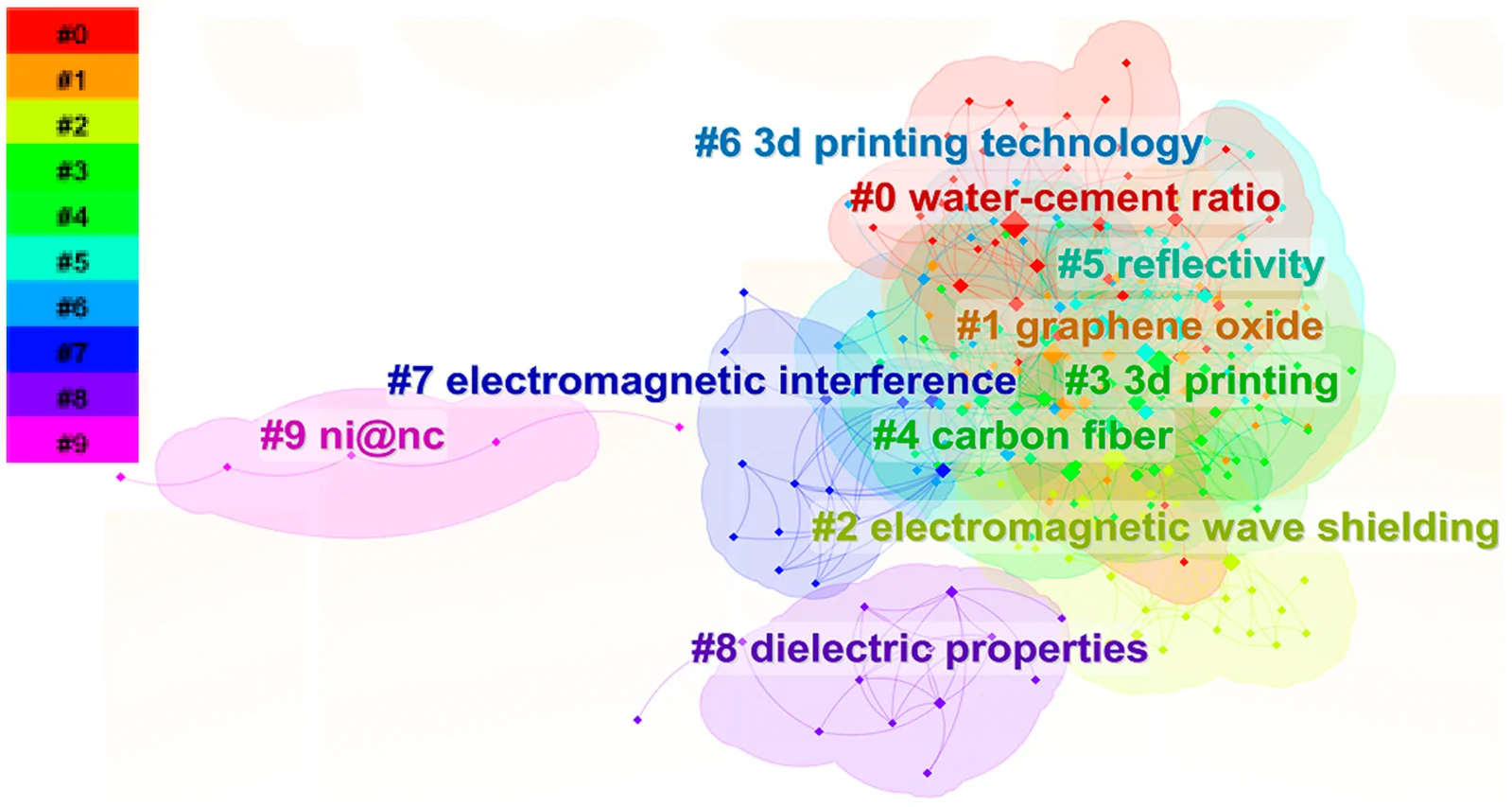
Keyword clustering map of cement-based wave-absorbing materials (2015–31 July 2025). Modularity Q = 0.4905; weighted mean silhouette S = 0.7469.

Using CiteSpace 6.4, we generated a keyword co-occurrence map for cement-based wave-absorbing materials from 2015 to 31 July 2025, as shown in Figure 5. The 10 most frequent keywords were: concrete (55), composites (48), microwave absorption (44), electromagnetic wave absorption (41), mechanical properties (38), cement-based composites (36), carbon nanotubes (32), graphene oxide (29), reflection loss (27), and shielding effectiveness (25). Synonymous keyword variants (e.g., microwave absorption, wave absorption, absorption, and absorbing property) were not fully merged before clustering; they were treated as equivalent terms in the interpretation. The keyword clustering map (Figure 6) classifies the keywords into distinct thematic clusters. The clustering quality was acceptable (Modularity Q = 0.4905; weighted mean silhouette S = 0.7469). Per-cluster size and silhouette values are reported in Table 4. The clustering algorithm identified ten core research clusters (#0–#9), covering the main research directions in this field from 2015 to 31 July 2025. Table 4 summarizes the technical meanings of these keyword clusters.

The keyword clusters become scientifically meaningful only when interpreted through the mechanism–design–performance cascade. Table 5 maps each cluster to its design strategy, absorption mechanism, representative performance, and critical bottleneck. Cluster #1 (graphene oxide) and #4 (carbon fiber) correspond to conductive-network and interfacial-polarization strategies [42,43]. CNT/cement composites show a transition from narrowband to broadband absorption as CNT loading increases from ~0.6 to ~0.9 wt.%, but only when dispersion is maintained; otherwise, agglomeration degrades both conductive loss and interfacial polarization [10]. In situ grown CNTs on slag can achieve an EMI shielding effectiveness of 55 dB over 8–12 GHz [31], while CNT periodic gratings achieve an average reflectivity of −15.5 dB over a 14.2 GHz bandwidth [44]. Magnetic clusters, including #9 (Ni@NC) and ferrite-related terms, correspond to natural resonance, exchange resonance, and eddy-current suppression [45]. Core–shell Fe_3_O_4_@SiO_2_ in magnesium phosphate cement exhibits an RLmin of −16.2 dB with an EAB of 1.2 GHz in the C-band and a compressive strength around 42 MPa [46]. Porous clusters map to impedance matching and multiple scattering. Foamed cement with 61.5% porosity achieves an EAB of 12.7 GHz but a compressive strength of only ~8 MPa [47], whereas foam with KH550@Fe_3_O_4_ resonant cavities reaches an EAB of 14.8 GHz at ~9 MPa [14]. Multilayer and 3D clusters correspond to impedance grading and geometric scattering [48]. Double-layer foamed cement/MWCNT achieves an RL of −47.3 dB and an EAB of 14 GHz [49], while pyramidal carbon-black mortar maintains >44 MPa with an RLmin of −40.3 dB [50].

Most studies in this corpus focus on laboratory-scale specimens rather than on prefabricated structural or finishing elements. Only a limited number of studies examine 3D-printed or multilayer cementitious components, and systematic application in concrete prefabrication remains rare. This gap between laboratory-scale specimens and prefabricated construction products is discussed further in Section 4.1.

### 3.6. Keyword Burst and Temporal Evolution of Technologies

**Figure 7:**
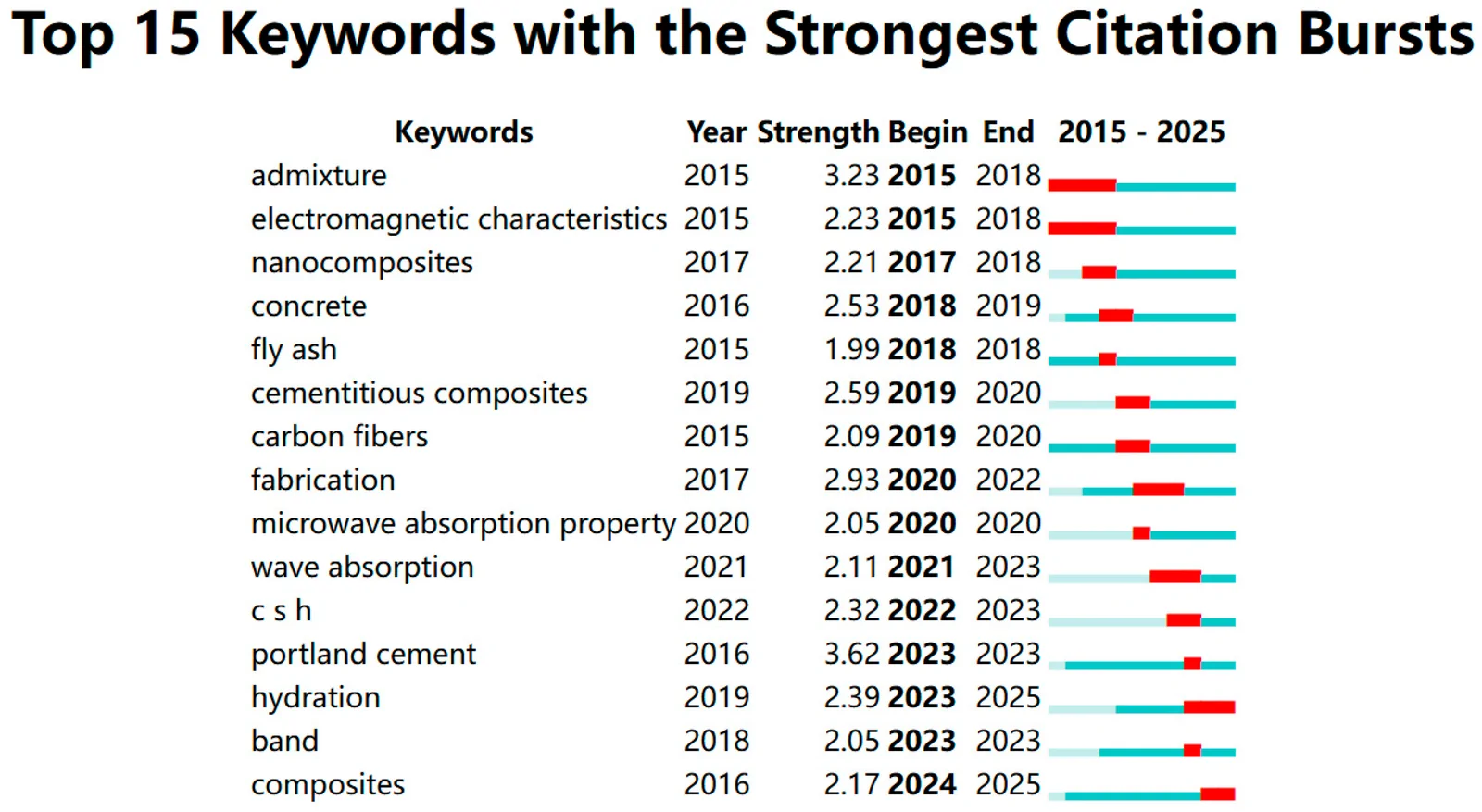
Top 15 keywords with the strongest citation bursts in cement-based wave-absorbing materials (2015–31 July 2025). Dark bars indicate burst periods. Keywords are ordered by burst start year; the strongest burst was ‘portland cement’ (strength = 3.62, 2023). The burst trajectory reveals three stages: admixture/matrix exploration, filler modification/system forming, and microscopic mechanism deepening.

Burst detection was conducted using CiteSpace’s default gamma parameter (γ = 1.0), with only keywords exceeding a burst strength of 1.5 retained. Figure 7 shows the top 15 keywords with the strongest citation bursts, ordered by burst start year. Based on burst onset/end times and intensity characteristics, the development trajectory from 2015 to 31 July 2025 was divided into three distinct phases. The first phase was the fundamental exploration stage of admixtures and matrix (2015–2018). “Admixture” and “electromagnetic characteristics” first exhibited bursts in 2015, followed by successive bursts of “fly ash” and “concrete.” Research during this phase centered on cement matrix modification strategies, employing mineral admixtures and various additives to tailor matrix formulations and investigate basic EM response characteristics [51]. The second phase was the filler modification and system formation stage (2019–2022). “Cementitious composites” and “carbon fibers” exhibited concentrated bursts, signaling that functional filler modification had become mainstream. Research focused on the synergistic optimization of mechanical and wave-absorbing properties through carbon fiber doping [52]. The sustained burst of “fabrication” keywords signaled a shift in research emphasis from simple filler doping ratios toward preparation process optimization and microstructural regulation [53]. The third phase was the stage of microscopic mechanism deepening and frontier exploration (2023–31 July 2025). Microscopic-scale keywords such as “hydration” and “C-S-H” exhibited concentrated bursts, signaling deeper investigation into the microstructural evolution of cement hydration products and the intrinsic relationships among hydration behavior, phase composition, and EM loss mechanisms [54,55,56,57,58].

Burst detection provides a complementary view of keyword dynamics. The first stage (2015–2018) was characterized by bursts of “admixture,” “fly ash,” and “concrete,” reflecting early exploration of matrix modification. The second stage (2019–2022) showed concentrated bursts of “cementitious composites” and “carbon fibers,” indicating a shift toward functional filler modification. The third stage (2023–31 July 2025) exhibited bursts of “hydration” and “C-S-H,” suggesting deeper investigation into microstructure–property relationships. These burst patterns are broadly consistent with the stage-wise trends described in Section 3.1. However, they serve here as a descriptive observation rather than as validation of the stage boundaries. The strongest burst was “portland cement” (strength = 3.62, burst year 2023), which reflects renewed attention to the cement matrix itself rather than to functional fillers alone. This suggests a shift toward understanding how hydration products and matrix chemistry govern EM loss, complementing the concurrent bursts of “hydration” and “C-S-H.”

### 3.7. Critical Synthesis: Mechanism–Design–Performance–Challenge Linkages

The bibliometric clusters summarized above are used here only as an organizing framework. The critical synthesis below focuses on materials strategies, absorption mechanisms, performance trade-offs, and engineering bottlenecks, drawing on representative experimental studies rather than on keyword counts. This section therefore carries the main materials-science conclusions of the review.

Table 5 maps each keyword cluster to its design strategy, absorption mechanism, representative performance, and critical bottleneck, converting bibliometric clusters into technical diagnostics. Table 6 then groups the representative systems by material strategy. Carbon-based conductive networks (CNT, graphene/GO) provide wide bandwidth compared with other compositional strategies, but they suffer from dispersion and impedance-mismatch problems. Foamed and 3D architectures can achieve even wider EAB, but at the expense of compressive strength or with greater fabrication complexity. Magnetic fillers such as Fe_3_O_4_@SiO_2_/MPC offer better impedance matching and higher strength but remain narrowband [46]. Porous and foamed systems achieve broadband absorption through multiple scattering and impedance grading, but at the cost of compressive strength [59]. Multilayer and 3D architectures can achieve broadband absorption with either high compressive strength (pyramidal CB mortar, >44 MPa) or lower flexural strength (3D printed element, ~13 MPa), but fabrication complexity remains high [50,60]. Waste-derived systems such as copper slag are low-cost but currently narrowband [61]. Key quantitative values for each strategy group are reported in Table 6; the text focuses on trade-offs rather than repeating individual data points.

This grouping shows that the field is not a collection of isolated material systems but a set of competing strategies with distinct trade-offs. Porous foams can achieve ultra-broadband absorption (EAB up to 14.8 GHz) but often with compressive strength <10 MPa [14], whereas pyramidal carbon-black mortar can maintain >44 MPa with broadband absorption [50], while 3D printed wave-shaped elements show lower flexural strength (~13 MPa) [60].

However, the bibliometric network also reveals that mechanism, forming, and durability studies remain weakly connected. This fragmentation hinders the translation of laboratory-scale absorbers into engineering practice.

To ground these gaps in the corpus rather than in interpretation alone, we screened the 292 records for three indicators. Of the 292 records, 15 mention at least one durability-related exposure (freeze–thaw, carbonation, chloride ingress, or aging); 17 report absorption data below 2 GHz; 38 report S/C-band (2–8 GHz) data; and 7 explicitly link 3D printing or additive manufacturing to loss mechanisms. Three knowledge gaps are proposed based on the combined bibliometric results, the corpus-level counts above, and a critical synthesis of representative studies: (i) limited research explicitly linking EM-loss mechanisms to advanced 3D-printing processes; (ii) insufficient attention to low-frequency performance and long-term durability; and (iii) internationally fragmented collaboration, observed as low network densities and limited cross-country co-authorship in the retrieved corpus. This fragmentation may hinder the development of standardized testing protocols. This link is interpretive: the bibliometric data indicate limited cross-border collaboration, but they do not directly demonstrate that standardization is constrained.

### 3.8. Quantitative Trade-Offs Across Absorption Strategies

The representative systems summarized in Table 6 are reorganized here by material strategy to address a practical question: which class of additives currently offers the most favorable balance of absorption, strength, cost, and durability? Four strategy classes are compared: CNT, graphene/GO, ferrite, and multilayer/3D architectures.

Ferrite-based systems, particularly Fe_3_O_4_@SiO_2_ in magnesium phosphate cement, show a favorable strength–absorption combination for structural applications, with an RLmin of approximately −16.2 dB and a 7-day compressive strength of approximately 42 MPa, although their EAB remains narrow (1.2 GHz over 2–18 GHz, RL < −10 dB). However, this assessment is based on a limited number of studies, and both magnesium phosphate cement and nano-Fe_3_O_4_ are more expensive than ordinary Portland cement. Ferrite/MPC should therefore not be described as a low-cost solution.

CNT/graphene systems provide wider bandwidth (e.g., an EAB of 7.1 GHz over 8–18 GHz for CNT/cement) but suffer from dispersion and cost penalties. Pyramidal carbon-black mortar achieves broadband absorption with high compressive strength (>44 MPa), whereas the 3D printed wave-shaped element achieves a wider EAB (17 GHz over 1–18 GHz) but with much lower flexural strength (~13 MPa). Fabrication complexity remains a limitation for both.

Carbonyl iron, biochar/waste-derived carbon, and MXene are not included in this comparison because the retrieved cement-based literature lacks sufficient RL/EAB, strength, and durability data for a fair assessment. This absence itself indicates an evidence gap in the field.

No single strategy simultaneously optimizes RL, strength, cost, and durability. The central engineering barrier is therefore no longer achieving RL < −20 dB in the laboratory, but sustaining absorption and mechanical integrity over long-term service exposure consistent with building service life (on the order of decades), including freeze–thaw cycles, carbonation, chloride ingress, and alkaline degradation of functional fillers.

## 4. Implications, Limitations, and Future Directions

### 4.1. Implications for Engineering Translation

The multi-scale perspective adopted here is qualitative and interpretive. It is intended to structure the critical synthesis rather than to propose a quantitative design methodology. The bibliometric results and critical synthesis in Section 3 reveal a clear transition from composition-centric modification to integrated material–structure co-design. However, this transition has not yet been matched by engineering translation. The keyword clusters show that mechanism, forming, and durability studies remain weakly connected, which explains why laboratory-scale absorbers rarely reach large-scale application. Three implications follow from this diagnosis.

First, future design must be organized around a multi-scale perspective rather than isolated material optimization. At the microscale, dielectric and magnetic loss mechanisms must be quantitatively decoupled under realistic conditions. At the mesoscale, filler dispersion, interfacial polarization, and pore architecture should be co-optimized with impedance matching. At the macroscale, multilayer and 3D gradient structures should be designed together with mechanical load-bearing requirements. Table 5 and Table 6 summarize representative strategies and performance trade-offs, showing that no single material system simultaneously optimizes bandwidth, strength, durability, and cost.

Second, the persistent trade-off between electromagnetic performance and mechanical integrity must be addressed through architecture rather than filler loading alone. Porous foams can achieve an effective absorption bandwidth (EAB) up to 14.8 GHz but often with compressive strength below 10 MPa, whereas multilayer and 3D gradient structures can maintain over 40 MPa with broadband absorption. This indicates that structural design is not an add-on but a primary route to break the EM–mechanical trade-off.

Third, engineering translation requires standardized testing protocols and durable performance evaluation. The country and institution collaboration networks had densities of 0.0521 and 0.0140, respectively, for the pruned networks used for visualization. These values are consistent with fragmented international collaboration. This fragmentation may limit the development of harmonized measurement methods, although the link is interpretive rather than directly demonstrated. Because density depends on network size and pruning, these values are not treated as standalone metrics.

Future engineering application should therefore prioritize internationally coordinated standardization, especially for low-frequency (S- and C-band) performance and coupled environmental loads such as freeze–thaw cycles, chloride ingress, and carbonation.

### 4.2. Limitations

Several limitations should be considered when interpreting these results. First, this bibliometric study was based on records retrieved from the Web of Science Core Collection; therefore, the results may have been influenced by database coverage and indexing policies. We included only original articles and review papers, excluding conference proceedings, meeting abstracts, editorials, and book chapters. As a result, outputs disseminated through conferences or non-article formats—particularly in engineering-oriented communities—may be underrepresented.

Second, only the Web of Science Core Collection was used; Scopus was not included. Although WoS provides consistent coverage for this field, records indexed solely in Scopus may be underrepresented.

Third, citation-based indicators are subject to time-lag effects and database indexing delays; therefore, we primarily focused on publication trends and collaborative patterns rather than citation-based metrics.

Fourth, the search string was not fully comprehensive. Variants such as “electromagnetic wave absorption,” “microwave absorbing,” “radar absorbing,” “wave absorption,” and “EMI” were not explicitly included, potentially omitting a small number of relevant records. Such omissions may affect keyword-based counts and cluster labels, although they are unlikely to change the overall publication trends and collaboration patterns.

Fifth, collaboration and productivity analyses rely on author names and affiliation information in bibliographic records. Institution and country names were not fully standardized (e.g., TURKEY/TURKIYE; China Building Materials Academy variants). In addition, synonymous keyword variants were not fully merged before clustering. These issues may slightly affect counts, network density, and cluster labels, although they do not change the main conclusions.

Sixth, network densities are reported for the pruned networks used for visualization; unpruned densities would be higher. Because density depends on network size and pruning, it is not used as a standalone indicator of collaboration strength. The link between fragmented collaboration and limited standardization is therefore interpretive rather than directly measured. We did not add the additional metrics suggested by the reviewer (proportion of internationally co-authored papers, number of connected components, or comparison with a reference field). Future work should incorporate these metrics to provide a more robust basis for gap (iii).

Seventh, 2025 is a partial year (data up to 31 July 2025). Publication counts and growth trends for 2025 may therefore be underestimated. Eighth, bibliometric analyses are primarily descriptive and correlational; therefore, external factors are discussed as contextual considerations rather than causal determinants. Finally, researchers tend to cite their own papers and high-impact publications; consequently, such papers and journals appear more frequently in citation and journal analyses.

### 4.3. Future Directions

To bridge the identified knowledge gaps and accelerate engineering translation, four future directions are proposed.

First, AI-guided inverse design should be tailored to cement-based systems. Unlike polymer or metal matrix composites, cementitious materials exhibit time-dependent hydration kinetics, an alkaline pore solution that can degrade functional fillers, and strong coupling between electromagnetic and rheological/mechanical properties. Future AI frameworks should therefore move beyond static property prediction to include multi-objective optimization that integrates EM absorption, mechanical strength, workability, and long-term durability. Hydration-aware modeling and alkaline durability prediction should also be incorporated. This is particularly relevant to the CNT dispersion and impedance-matching bottlenecks summarized in Table 5.

Second, additive manufacturing (AM) of cement-based absorbers should exploit cement-specific rheological and hydration characteristics. Extrusion-based 3D printing offers unique opportunities for functionally graded metamaterials, including in situ compositional grading, filler alignment control, and interlayer interfaces as additional polarization sites. However, the printability–functionality trade-off must be carefully managed, because functional fillers dramatically alter yield stress and thixotropy. AI-assisted printable window prediction and rheology modifiers can help balance extrudability and electromagnetic performance. This directly addresses the weak coupling between EM-loss mechanisms and forming processes identified in Section 3.7.

Third, durability-oriented standardization is urgently needed. Long-term performance under coupled environmental loads—freeze–thaw cycles, chloride ingress, sulfate attack, and elevated temperatures—remains largely unquantified. International joint standardization projects involving leading institutions from China, Australia, Europe, and other regions should be initiated to harmonize measurement methods and develop benchmark reference materials. This would simultaneously enhance global collaboration density and facilitate industry standards. This responds to the fragmented collaboration and the non-comparable thickness and frequency bands evident in Table 6.

Fourth, sustainable material cycles should be integrated into design. Industrial by-products such as copper slag, fly ash, and secondary aluminum ash can serve as functional fillers or foaming agents, providing a pathway to balance the cost–performance trade-off. However, their long-term stability and environmental impact must be systematically evaluated. This concern also applies to engineered nanofillers. Recent evidence shows that nanofillers incorporated in cementitious matrices can leach under environmental exposure; their aquatic ecotoxicity and environmental fate must therefore be assessed alongside electromagnetic performance and mechanical durability [62]. Such leaching behavior is particularly relevant for cement-based absorbers exposed to rainfall, freeze–thaw cycles, and carbonation. Coupling waste valorization with multi-scale design and AI optimization could therefore yield low-cost, high-performance, and environmentally adaptable cement-based absorbers. This potential can be realized only if long-term stability and environmental impact are explicitly included in the design loop.

In summary, the field is moving from empirical formulation to mechanism-guided, data-driven, and architecture-enabled design. Realizing this vision requires sustained collaboration across materials science, electromagnetics, civil engineering, and data science, positioning cement-based composites at the heart of next-generation spectrally aware and responsive built environments.

## 5. Conclusions

What did we learn? First, no single absorption strategy simultaneously optimizes RL, strength, cost, and durability. Among the systems reviewed, ferrite/MPC offers a favorable strength–absorption combination, but not necessarily the best cost balance; its raw materials are not low-cost, and the evidence base is limited to a small number of studies. CNT/graphene systems provide wider bandwidth at the expense of higher cost and greater dispersion challenges. Second, the dominant barrier is no longer achieving RL < −20 dB in the laboratory; it is sustaining absorption and mechanical integrity over long-term service exposure consistent with building service life (on the order of decades). Third, priority should shift from incremental filler optimization to durability-oriented standardization and cement-specific design.

The bibliometric analysis provides supporting context for these materials-science conclusions. It analyzed 292 Web of Science records from 2015 to 31 July 2025 and identified three growth stages: embryonic exploration (2015–2018), steady growth (2019–2022), and rapid expansion (2023–31 July 2025). China contributed 175 papers (60.0%), but the country collaboration density was only 0.0521, suggesting fragmented international collaboration. Keyword and cluster analyses revealed a shift from admixture and matrix modification to carbon/magnetic filler engineering, porous and multilayer architectures, 3D printing, hydration products, and microstructure regulation. These patterns frame, rather than replace, the materials-science lessons above.

These materials-science lessons, together with the corpus-level counts reported in Section 3.7, point to three knowledge gaps: (i) weak coupling between EM-loss mechanisms and advanced 3D-printing processes; (ii) insufficient attention to low-frequency performance and long-term durability; and (iii) internationally fragmented collaboration that may hinder the development of standardized testing protocols. The bibliometric findings are supported by data. By contrast, the three knowledge gaps are interpretive proposals derived from these findings and from the representative studies cited in Section 3.7.

Future research should link forming-process variables with EM-loss mechanisms and initiate international standardization projects. It should also strengthen durability studies under coupled environmental loads and adopt AI-guided inverse design and cement-specific additive manufacturing. These directions follow directly from the three lessons above and could make cement-based wave-absorbing materials more efficient, durable, and scalable for next-generation built environments.

## Figures and Tables

**Table 1:** Stage-wise technological evolution and bottlenecks of cement-based wave-absorbing materials.

Stage	Publication Trend	Technological Breakthrough	Representative System	Performance	Remaining Bottleneck
2015–2018	3–11/year	Admixture and matrix modification	Fly ash, carbon black	Basic EM testing	No systematic mechanism–property framework
2019–2022	15–32/year	Carbon-based filler networks	CNT, GO, CF, ferrite	RL −28 dB, EAB 7.1 GHz	Dispersion, impedance mismatch
2023–31 July 2025	36–78/year	Multi-scale, porous, multilayer, 3D printing	Foam, multilayer, gradient	EAB 14.8 GHz, >40 MPa	Durability, scalability, standardization

**Table 2:** Top five countries/regions in publication output and their representative topics.

Ranking	Country/Region	Number of Published Papers	Representative Topics
1	China	175	Carbon fillers, porous/multilayer, 3D printing
2	USA	28	EMI shielding, metamaterials
3	India	15	Ferrites, dielectric absorbers
4	South Korea	14	CNT/cement, EMI shielding
5	Australia	13	3D concrete printing, structural absorbers

Note: Representative topics are derived from keyword frequency within each country/region group in the 292-record corpus. For each country, the two to three most frequent keywords are listed.

**Table 3:** Top 10 research institutions and their representative research profiles.

Ranking	Institution	Country/Region	Publications	Representative Keywords/Clusters
1	Hebei University of Technology	China	20	Carbon fillers, EM parameter regulation
2	Wuhan University of Technology	China	17	GO-modified cement, 3D printing
3	Dalian University of Technology	China	14	CF/cement, EMI shielding
4	China Building Materials Academy	China	13	Carbon black/cement, porous glass
5	Shenyang Ligong University	China	12	Ferrite/graphite, multi-scale design
6	University of Western Australia	Australia	11	3D concrete printing
7	Xi’an University of Architecture and Technology	China	10	Multilayer absorbers
8	Edith Cowan University	Australia	9	Structural–functional integration
9	Korea Advanced Institute of Science and Technology (KAIST)	South Korea	8	CNT/cement, EMI shielding
10	Southeast University	China	8	Cement-based composites

Note: Representative keywords were derived from keyword frequencies within each institution group in the 292-record corpus. For each institution, the two to three most frequent keywords are listed.

**Table 4:** Summary of keyword clustering for cement-based wave-absorbing materials (2015–31 July 2025).

Cluster	Original LLR Label	LLR Top Terms	Size	Silhouette	Final Label Used in Text	Technical Meaning
#0	water–cement ratio	water–cement ratio; pore structure; dielectric loss; magnetic loss	42	0.76	matrix and pore engineering	Matrix and pore engineering
#1	graphene oxide	graphene oxide; dielectric fillers; reflection loss; impedance matching	36	0.81	graphene oxide	Carbon filler modification
#2	electromagnetic wave shielding	shielding effectiveness; structural regulation; EMI shielding	31	0.74	electromagnetic wave shielding	Application scenarios
#3	3D printing (merged with #6)	3D printing; additive manufacturing; 3D concrete printing; hollow glass microspheres	58	0.72	3D printing	Advanced forming and metamaterial-inspired design
#4	carbon fiber	carbon fiber; carbon black; slag; conductive network	29	0.79	carbon fiber	Conductive network
#5	reflectivity	copper slag; cement paste-resin; micro-gradient; reflection loss	25	0.73	micro-gradient and reflectivity	Performance evaluation
#7	electromagnetic interference	EMI shielding; radiation absorption; building shielding	23	0.77	electromagnetic interference	Building shielding
#8	dielectric properties	pore structure; conductivity; XRD; SEM; microstructure	20	0.75	microstructure–property relation	Microstructure–property relation
#9	ni@nc	MOF-derived; CNT nanocomposites; magnetic loss; polarization	18	0.78	Ni@NC	Magnetic–dielectric hybrid

Note: Original LLR labels and top terms are exported from CiteSpace. Cluster #6 (“3D printing technology”) was merged into #3 because both clusters share 3D-printing-related terms; the table therefore lists nine clusters, and the article refers to nine clusters in total. Size and silhouette values are from the CiteSpace cluster summary. Final labels are used consistently in Table 5, Section 3.5 and Section 3.7, and Figure 6.

**Table 5:** Keyword cluster–design–mechanism–performance–challenge mapping.

Cluster	Design Strategy	Mechanism	Representative Performance	Critical Bottleneck
#0 water–cement ratio	Matrix/pore engineering	Impedance matching, polarization	Permittivity tuning	Durability, reproducibility
#1 graphene oxide	Carbon filler modification	Conductive loss, interfacial polarization	GNPs + EMD EAB 5.8 GHz	Dispersion, impedance mismatch
#2 EM shielding	Application scenario	EMI shielding	Shielding effectiveness	Standard testing
#3 3D printing (merged with #6)	Advanced forming and metamaterial-inspired design	Impedance grading; multi-resonance	3D printed wave-shaped RL −43.7 dB, >40 MPa; FCAM RL −56.59 dB	Scalability; fabrication complexity
#4 carbon fiber	Conductive network	Conductive loss, scattering	CF/cement EMI shielding	EM–mechanical trade-off
#5 reflectivity	Performance evaluation	Reflection loss	RLmin −40 dB	Low-frequency
#7 EMI	Building shielding	EMI shielding	SE 55 dB	Long-term durability
#8 dielectric properties	Microstructure	Dielectric loss	Permittivity tuning	Mechanism decoupling
#9 Ni@NC	Magnetic–dielectric hybrid	Magnetic loss, polarization	RL −45.76 dB	Alkaline stability

Note: Cluster #6 (“3D printing technology”) was merged into #3 because both clusters share 3D-printing-related terms. The table therefore lists nine clusters, consistent with Table 4. The final cluster labels are identical to those in Table 4 and are used consistently throughout the text.

**Table 6:** Comparison of representative cement-based EMW-absorbing composites and their performance trade-offs.

Strategy Group	System	Thickness	Frequency Band	Method/RL Type	EAB	Relative Bandwidth	EAB/Thickness	RLmin	**Strength**	**Key Limitation**
Carbon-based conductive network	MWCNT/cement [10]	25 mm	2–18 GHz	NRL arc/Measured	7.1 GHz	0.443	0.284	−28 dB	~35 MPa	Narrowband
	CNT/cement [37]	20 mm	8–12.5 GHz	NRL arc/Measured	4.5 GHz	1.0	0.225	−14.5 dB	~42.8 MPa	Dispersion
	GNPs + EMD [42]	10 mm	2–18 GHz	NRL arc/Measured	5.8 GHz	0.36	0.580	−21.6 dB	~39.7 MPa	Impedance matching
Magnetic fillers	Fe_3_O_4_@SiO_2_/MPC [46]	20 mm	2–18 GHz	NRL arc/Measured	1.2 GHz	0.075	0.06	−16.2 dB	~42 MPa	Narrowband
Porous/foamed systems	Foamed cement [47]	40 mm	2–18 GHz	NRL arc/Measured	12.7 GHz	0.793	0.318	~−25 dB	<13.5 MPa	Low strength
	Foam + KH550@Fe_3_O_4_ [14]	25 mm	2–18 GHz	NRL arc/Measured	14.8 GHz	0.925	0.592	~−38 dB	~9 MPa	Low strength
Multilayer/3D architectures	Double-layer foam/MWCNT [49]	30 mm	2–18 GHz	NRL arc/Measured	14 GHz	0.875	0.466	−47.3 dB	7 MPa	Low strength
	Three-layer EPS/CB [48]	70 mm	1–18 GHz	NRL arc/Measured	14.3 GHz	0.841	0.204	−49.6 dB	<1 MPa	Low strength
	Pyramidal CB mortar [50]	30 mm	1–18 GHz	NRL arc/Measured	~16.9 GHz	1.0	0.563	−40.3 dB	>44 Mpa	Complex fabrication
	3D printed wave-shaped [60]	45 mm	1–18 GHz	NRL arc/Measured	~17 GHz	1.0	0.377	−37.4 dB	13 MPa (Flexural)	Additive manufacturing
Waste-derived systems	Copper slag [61]	10 mm	8.2–12.4 GHz	NRL arc/Measured	0.7 GHz	0.166	0.07	−13.4 dB	40.6 MPa	Narrowband

Note: Strength values are compressive unless marked “flexural.” EAB is the frequency range with RL < −10 dB. Because tested frequency windows differ, EAB values are compared using relative bandwidth (EAB/tested-window width) and EAB/thickness. Table 6 includes only strategy groups with sufficient quantitative RL/EAB and strength data; waste-derived and shielding-only studies were not included because of insufficient comparable data. Representative studies were selected if they (i) report quantitative RL/EAB or SE; (ii) specify thickness and tested frequency window; (iii) represent a strategy group; (iv) are from the 292-record corpus; and (v) preferably report strength or durability data. up to four representative studies for the multilayer/3D group are shown to illustrate trade-offs, not to be exhaustive.

## Data Availability

The search string, Web of Science categories, screening criteria, exclusion counts, and CiteSpace parameter settings are reported in Section 2.1 and Section 2.2. The bibliographic records and the CiteSpace project file are available from the corresponding author upon reasonable request, subject to the Web of Science license terms. No new data were created or analyzed in this study. Data sharing is not applicable to this article.

## Supplementary Materials

The following supporting information can be downloaded at: https://www.mdpi.com/article/10.3390/ma19214542/s1, Figure S1: PRISMA 2020 flow diagram showing the identification, screening, and inclusion of records.
